# Editorial: Targeted next-generation sequencing for pathogen and antimicrobial resistance (AMR) identification and profiling

**DOI:** 10.3389/fcimb.2025.1633941

**Published:** 2025-06-27

**Authors:** Sandip Patil, Feiqiu Wen, Alfizah Hanafiah, Prakash M. Halami

**Affiliations:** ^1^ Department of Haematology and Oncology, Shenzhen Children’s Hospital, Shenzhen, China; ^2^ Paeditaric Research Institue, Shenzhen Children’s Hospital, Shenzhen, China; ^3^ Departement of Medical Microbiology & Immunology, Faculty of Medicine, Universiti Kebangsaan Malaysia, Kuala Lumpur, Malaysia; ^4^ Microbiology & Fermentation Technology, Central Food Technological Research Institute (CFTRI), Mysore, India

**Keywords:** next-generation sequencing, antimicrobial resistance, pathogen genomics, zoonotic transmission, targeted therapy

The global threat of antimicrobial resistance (AMR) demands innovative and precise diagnostic strategies that can keep pace with evolving pathogens. Among such approaches, targeted next-generation sequencing (NGS) has emerged as a transformative tool in infectious disease diagnostics, offering unprecedented resolution in pathogen identification and resistance gene profiling. This Research Topic of *Frontiers in Cellular and Infection Microbiology* brings together pioneering studies that showcase the clinical, genomic, and translational power of targeted NGS across diverse infectious contexts.

In a clinical setting, Li et al. investigated the diagnostic utility of nanopore-targeted sequencing (NTS) using bronchoalveolar lavage fluid (BALF) from patients with pulmonary infections. NTS demonstrated significantly higher sensitivity than conventional microbial testing (CMT) (86.13% vs. 67.15%) without compromising specificity (Importantly, it maintained diagnostic performance in antibiotic-pretreated patients—an area where traditional diagnostics often fail. Therapeutic regimens guided by NTS led to marked clinical improvements, supporting its real-time clinical relevance and impact on decision-making. Expanding the scope of respiratory infections, Huang et al. used NGS to characterize microbial profiles in patients with non-small-cell lung cancer (NSCLC) undergoing sleeve lobectomy. They uncovered a pattern of shared dominant bacteria (*Streptococcus pneumoniae*, *S. pseudopneumoniae*, *Haemophilus parainfluenzae*) in patients with and without obstructive pneumonia. Interestingly, human herpesvirus 7 was enriched in patients with prolonged postoperative drainage, highlighting the utility of NGS in deciphering host-microbiome interactions that may influence surgical outcomes and recovery. Addressing the burden of AMR in vulnerable populations, Chen et al. presented a comprehensive clinical and genomic analysis of carbapenem-resistant Enterobacterales (CRE) bloodstream infections (BSIs) in patients with hematologic malignancies. Whole-genome sequencing of 45 CRE isolates revealed the dominance of high-risk clones—*Klebsiella pneumoniae* ST11 (*bla*_KPC-2_) and *Escherichia coli* harbouring *bla*_NDM-5_. CRE BSIs were associated with high mortality (56.9% 30-day mortality), highlighting the urgency for genomic surveillance and tailored therapeutic strategies in immunocompromised hosts. Tagueha et al. offered a longitudinal genomic perspective, characterizing a decade’s evolution of carbapenem-resistant *Acinetobacter baumannii*. They noted phenotypic divergence over time: older isolates showed greater desiccation tolerance and urinary tract affinity, while recent respiratory isolates exhibited increased biofilm formation. This shift underscores the need for dynamic genomic monitoring to inform infection control and predict shifts in clinical manifestations.

Zoonotic transmission and environmental reservoirs were explored by Wu et al., who investigated the epidemiology of monophasic *Salmonella Typhimurium* ST34 (mSTM ST34). Their genomic analysis revealed rapid global dissemination and replacement of the previously dominant ST19 lineage. Notably, mSTM ST34 exhibited high multidrug resistance—facilitated by IncQ1 plasmids—and traits enhancing colonization and biofilm formation. The study raises concerns about treatment failure risks and supports the development of alternative therapies, such as phage-based interventions. Further emphasizing the value of NGS in critical care, Wang et al. applied metagenomic profiling and latent class analysis to stratify immunocompetent patients with severe community-acquired pneumonia (sCAP) requiring invasive ventilation. They identified two distinct clinical phenotypes with differing responses to corticosteroid treatment. Notably, corticosteroids significantly reduced mortality only in one phenotype, suggesting that rapid stratification based on metagenomic data could guide personalized immunomodulatory therapy.

In a neonatal context, Zhou et al. reported an outbreak of CRKP in a NICU. Whole-genome sequencing revealed multidrug resistance mechanisms including *mcr-1*, *bla*_NDM-1_, and *bla*_KPC-2_, alongside extensive plasmid diversity. The detection of multiple sequence types within a confined setting underscored the rapid spread and complexity of resistance determinants in neonatal care, calling for aggressive infection control and continuous genomic monitoring. Finally, Zhou et al. explored early microbiological response in ventilator-associated lower respiratory tract infections (VA-LRTIs) using quantitative targeted amplicon-based NGS (QtNGS). They demonstrated that changes in the relative quantification ratio (RQR) of *Acinetobacter baumannii* could accurately predict patient survival. Patients with RQR ≥1.41 had a significantly higher mortality risk, establishing RQR as a prognostic biomarker and reinforcing QtNGS as a tool for early therapeutic evaluation.

Together, these contributions highlight how targeted NGS is reshaping the landscape of infectious disease research and clinical practice. By merging genomic data with clinical insights, researchers are illuminating pathways of resistance evolution, uncovering novel diagnostic biomarkers, and informing personalized treatment strategies. As the field matures, integrating NGS into routine clinical workflows will be essential for enhancing antimicrobial stewardship, improving outcomes, and safeguarding public health against emerging microbial threats. We extend our sincere thanks to the contributing authors, peer reviewers, and editorial team for their dedication to advancing the science and application of targeted NGS. The studies presented in this Research Topic not only deepen our understanding of pathogen biology and AMR but also pave the way for the next generation of precision diagnostics and evidence-based interventions.

## Conclusion

The studies compiled in this Research Topic collectively demonstrate the transformative impact of targeted NGS in infectious disease diagnostics, surveillance, and management. From improving diagnostic sensitivity in complex pulmonary infections and stratifying pneumonia phenotypes to tracing AMR evolution in hospital outbreaks and characterizing high-risk pathogens in vulnerable populations, NGS has proven to be a critical tool in our fight against microbial threats. Importantly, these investigations illustrate how integrating genomic data with clinical and phenotypic insights can not only enhance patient care but also inform public health strategies and antimicrobial stewardship. As antimicrobial resistance continues to escalate and pathogen behaviour becomes more unpredictable, the implementation of targeted NGS into routine clinical workflows is no longer optional—it is essential. We hope this Research Topic of cutting-edge research will inspire further innovation, collaboration, and adoption of genomic technologies to improve diagnostic precision, optimize therapy, and ultimately protect global health.

